# Supplementary material for: The Systems Biology Research Tool: evolvable open-source software
Source: BMC Syst Biol. 2008 Jun 29;2:55. doi: 10.1186/1752-0509-2-55 (PMC2446383; doi:10.1186/1752-0509-2-55)
Supplement: Additional file 1 — SBRT Archive. An archive of the current version of the Systems Biology Research Tool. [file 1752-0509-2-55-S1.zip › sbrt-1.4.0/doc/developers_guide/api/sbrt/kernel/math/LinearComb.Term.html]

LinearComb.Term


|  |  |  |
| --- | --- | --- |
| **Overview** \| **Package** \| **Class** \| **Use** \| **Tree** \| **Deprecated** \| **Index** \| **Help** | |  |
| **PREV CLASS**   **NEXT CLASS** | **FRAMES**    **NO FRAMES**     **All Classes** |  |
| SUMMARY: NESTED \| FIELD \| CONSTR \| METHOD | DETAIL: FIELD \| CONSTR \| METHOD |  |


---


## sbrt.kernel.math Interface LinearComb.Term<V>

**Type Parameters:**: `V` - the variable type.

**All Superinterfaces:**: java.lang.Iterable<V>, Polynomial.Term<V>

**All Known Implementing Classes:**: AbstractLinearComb.Term, HashLinearComb.Term

**Enclosing interface:**: LinearComb<V>

---

``` public static interface LinearComb.Term<V> extends Polynomial.Term<V> ```

This interface is used to represent the terms of linear combinations.
A term contains a variable and a coefficient.

**Author:**
:   This interface was written and documented by Jeremiah Wright
    while in the Wagner lab.

---

| **Method Summary** | |
| --- | --- |
| `V` | `getVariable()`             Returns the variable contained in this term. |

**Methods inherited from interface sbrt.kernel.math.Polynomial.Term**

`equals, getCoefficient, getVariables, hasAllVariables, hashCode, hasVariable, iterator, power, toString, variables`

**Method Detail**

### getVariable

```
V getVariable()
```

:   Returns the variable contained in this term.

    :   **Returns:**: the variable contained in this term.


---


|  |  |  |
| --- | --- | --- |
| **Overview** \| **Package** \| **Class** \| **Use** \| **Tree** \| **Deprecated** \| **Index** \| **Help** | |  |
| **PREV CLASS**   **NEXT CLASS** | **FRAMES**    **NO FRAMES**     **All Classes** |  |
| SUMMARY: NESTED \| FIELD \| CONSTR \| METHOD | DETAIL: FIELD \| CONSTR \| METHOD |  |


---
